# Simulation of, Optimization of, and Experimentation with Small Heat Pipes Produced Using Selective Laser Melting Technology

**DOI:** 10.3390/ma16216946

**Published:** 2023-10-29

**Authors:** Jianfeng Zhou, Lai Teng, Yinyi Shen, Zhonghe Jin

**Affiliations:** 1 Micro-Satellite Research Center, Zhejiang University, Hangzhou 310007, China; 12224032@zju.edu.cn; 2 Huanjiang Laboratory; Shaoxing 311899, China; 21924085@zju.edu.cn; 3 Key Laboratory of Micro-Nano Satellite Research of Zhejiang Province, Hangzhou 310058, China; jinzh@zju.edu.cn

**Keywords:** microsatellite, heat pipe, additive manufacturing technology, SLM, multi-objective optimization

## Abstract

With the development of microsatellite technology, the heat generated by onboard components is increasing, leading to a growing demand for improved thermal dissipation in small satellites. Metal powder additive manufacturing technology offers the possibility of customizing and miniaturizing heat pipes to meet the specific requirements of small satellites. This article introduces a small-scale heat pipe designed using selective laser melting (SLM) technology. The heat pipe’s material, structure, and internal working fluid were determined based on mission requirements. Subsequently, the SolidWorks 2021 software was used for heat pipe modeling, and the ANSYS 2021R2 finite element analysis software was employed to simulate the heat transfer performance of the designed heat pipe, confirming its feasibility. The heat pipe’s structure was optimized using multi-objective regression analysis, considering various structural parameters, such as the channel diameter, vapor chamber height, and narrow gap width. The simulation results demonstrate that the optimized heat pipe achieved a 10.5% reduction in thermal resistance and an 11.6% increase in equivalent thermal conductivity compared to the original heat pipe. Furthermore, compared to conventional metal heat-conducting rods, the optimized heat pipe showed a 38.5% decrease in thermal resistance and a 62.19% increase in equivalent thermal conductivity. The heat pipe was then fabricated using a 3D printer (EOS M280), and a vacuum experimental system was established to investigate its heat transfer characteristics. The experimental results show that the heat pipe operated most efficiently at a heating power of 20 W, reached its maximum heat transfer capacity at 22 W, and had an optimal fill ratio of 30%. These results highlight the excellent performance of the heat pipe and the promising application prospects for SLM technology in the field of small satellites.

## 1. Introduction

With the continuous advancement of space technology, microsatellites have gradually become a research hotspot in the satellite industry due to their small size, low cost, and high innovation potential. Satellites in space face a complex and diverse thermal environment, with temperatures ranging from a minimum of 100 K to a maximum of 334 K. Therefore, maintaining stable internal temperatures through thermal control systems is essential to ensure the normal operation of satellites, extend the lifespan of expensive onboard instruments, and reduce overall project costs. This has made thermal control systems a major focus of research in satellite technology [1]. Indeed, there have been historical instances in which thermal control system anomalies have led to satellite failures. Examples include the Japanese “Osumi” satellite and a Canadian communications satellite. In both cases, thermal control system malfunctions resulted in uncontrolled internal instrument temperatures, ultimately causing damage to the satellites and significant losses. These incidents underscore the critical importance of robust and reliable thermal control systems in satellite design and operation to prevent such failures and safeguard expensive satellite payloads [2]. At present, the primary means of thermal dissipation for the satellites launched by our laboratory are thermal control coatings and metal thermal strips. However, with the increasing integration of electronic components and the growing number of high-power devices, there is an urgent need for a more efficient thermal control element. Therefore, this paper focuses on the design and study of heat pipes that can be used in microsatellites.

Many other research institutions also have similar requirements and have initiated studies on heat pipes. D. Khrustalev and A. Faghri, for instance, have researched heat pipe evaporator and condenser thin-film heat transfer models [3,4]. They established a mathematical model for low-temperature axial grooved heat pipes, taking into account the effects of the heat transfer in the liquid film inside the heat pipe channels, the heat transfer on the ribbed surfaces between channels, and the shear forces between the gas and liquid phases. The calculation results indicate that, as the heat load increases, the thermal resistance in the evaporator section of the heat pipe decreases, while the thermal resistance in the condenser section increases. Additionally, curvature changes primarily occur near the liquid plug region. Zhang Chengbin et al. established a theoretical model for “Ω”-shaped groove heat pipes and analyzed the flow and heat transfer inside. Using this model, they calculated the maximum heat transfer capacity of the heat pipe under different operating conditions, compared the results with the thermal management theoretical model established by Chi, and conducted experimental tests to validate the accuracy of this model. Furthermore, they employed genetic algorithms to study the optimal structural parameters of small heat pipes based on this model, optimizing the heat pipe’s structure to enhance its maximum heat transfer capacity [5,6]. Yao Fan et al. [7] established an axial grooved heat pipe model and experimentally and numerically assessed the impact of changes in the heating and heat transfer sections on the heat pipe’s performance. They also compared the heat transfer performance of heat pipes under the influence of gravity to those in a microgravity environment. The experiments showed that an increase in heating length can reduce thermal resistance, and heat pipes assisted via gravity exhibited superior performance .

With the continuous development of computer technology and the growing maturity of simulation software, finite element analysis can now be performed with grids ranging from millions to tens of millions, making it more efficient and accurate in depicting the flow and heat transfer characteristics of heat pipes compared to traditional methods. In recent years, many researchers have utilized finite element simulation techniques for more precise studies of heat pipes. Annamalai and Ramalingam conducted simulations of heat pipes using ANSYS 2021R2 software [8]. They assumed that there exists only a gas-phase monophasic state in the heat pipe’s vapor cavity and only a liquid-phase monophasic state in the wick core, excluding the evaporation and condensation phase change cycle. Through simulation, they analyzed the temperature distribution on the heat pipe’s outer wall and the internal vapor temperature, obtaining results that closely matched experimental data. Szczukiewicz et al. conducted simulation and experimental research on multiphase flow in heat pipes, specifically focusing on evaporation and condensation [9]. They analyzed local heat transfer coefficients and validated the rationality of the liquid film thermal inertia model between bubbles and channel walls. Rabiee et al. combined the analysis of evaporation and condensation phase-change heat transfer processes in closed-loop heat pipes [10]. They performed simulation and experimental research on U-shaped horizontal aluminum alloy heat pipes, considering the coupling effects of evaporation and condensation in a sealed container. The research results indicated that the model could effectively simulate phase-change characteristics in the heat pipe under different operating conditions. Furthermore, using simulation models, the independent parameters affecting heat pipe thermal resistance, such as heat input, filling ratio, pipe diameter, and working fluid type, were studied. Yosuke Yasuda and his team designed numerous heat pipes with varying cross-sectional shapes. They utilized simulation calculations to analyze the internal flow characteristics of the heat pipes, investigating their heat transfer performance. The research also examined the maximum power at which the heat pipes operate. Ultimately, the results indicated that changes in shape had a minimal impact on the heat transfer capability when the power reached 80 W [11]. Cristiano Enke and his team developed a mathematical model for an aluminum–ammonia axially grooved heat pipe. They used numerical simulations to simulate the internal flow within the heat pipe. In addition, they conducted experiments to investigate the impact of non-condensing gases inside the heat pipe on its heat transfer performance [12]. 

Experiments serve as a vital means to validate simulation and theoretical calculations, and many scholars have contributed valuable insights in this regard. Anand, considering axial gas–liquid interface shear forces and radial gas–liquid radius variations, established a heat transfer model for an aluminum–methane groove heat pipe. They then fabricated the physical heat pipe and conducted experiments under various conditions to validate the accuracy of the simulations and calculations [13]. Chao Shen proposed a flat parallel-flow heat pipe (FPFHP) based on gravity heat pipes and established an experimental platform to explore the effects of a heat pipe’s inner diameter, inclination angle, heating power, and fluid type on the heat pipe’s performance [14]. Wang Gang conducted experiments on heat pipes with various channel shapes to measure and analyze their heat transfer performance, as reported in reference [15]. The results indicate that at α = 0°, the FPMHP exhibits weaker heat performance because condensate flowback is solely driven by capillary forces. However, at α = 90°, the A-4 type FPMHP demonstrates the best performance, benefiting from the combined effects of capillary forces, gravity, and the working fluid volume. Hanping Che designed and manufactured a stepped wavy-finned tube with two layers of interleaved fins on the spiral groove sidewall of the tube surface [16]. Through experiments, its heat transfer capability was measured, and the results indicate that SLFT exhibits excellent heat transfer enhancement performance under both condensing and boiling conditions. This presents a promising solution for highly efficient, compact, integrated refrigeration and heating exchangers.

This study addresses the underexplored intersection of additive manufacturing technology (3D printing technology) and heat pipe utilization, particularly in the context of small satellites. We designed a heat pipe tailored for SLM technology, accommodating the specific needs of our laboratory’s small satellite projects. Our approach involved three-dimensional modeling to assess heat transfer performance, complemented by experimental validation, highlighting the viability of our design. Furthermore, we conducted a multi-objective optimization of the heat pipe’s structure, considering factors like evaporator–condenser proportions, groove diameter, vapor cavity height, and narrow gap width. This optimization led to a heat pipe structure surpassing the original design by 10% in heat transfer efficiency. Compared to conventional heat-conducting bars in our institute’s thermal control system, our heat pipe exhibited a 38% reduction in thermal resistance and a 62.19% increase in equivalent thermal conductivity.

## 2. Design of Heat Pipes

### 2.1. The Working Principle of Heat Pipes

**Figure 1:**
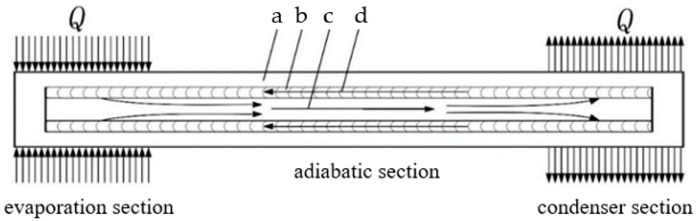
Schematic diagram of the working mechanism of heat pipes: (**a**) heat pipe shell; (**b**) channel; (**c**) working steam; and (**d**) working fluid.

Heat pipes are a commonly used and efficient heat transfer component in aerospace, with advantages such as high heat transfer performance, good temperature uniformity, strong adaptability, high reliability, and a fast start-up speed. These advantages have been attributed to their efficient and direct working principle. During the operation of a heat pipe, the evaporation section is subjected to an increase in the heat load and heats up the working fluid inside the pipe, causing the steam pressure in the evaporation section to be higher than that in the condensation section. The resulting pressure difference causes the gas-phase working fluid to flow towards the condensation section. The capillary pressure difference in the condensation section provides the power for the circulating flow of liquid-phase working fluid, which transfers heat energy from the evaporation section to the condensation section through the phase-change heat transfer of the working fluid in the heat pipe, achieving a faster and more stable heat transfer. The principle is shown in Figure 1.

According to the flow direction of the working fluid in Figure 1, it can be deduced that the vapor pressure in the evaporator section is greater than that in the condenser section, while the liquid-phase pressure in the condenser section is greater than that in the evaporator section. To ensure the stability of heat pipe operation, it is essential to ensure that the capillary pressure differential is greater than the sum of the liquid-phase pressure differential and the gas-phase pressure differential. This relationship is expressed as follows:

(1) $$∆P_{c}\geq ∆P_{l}\pm ∆P_{v}$$

The calculation formula for capillary pressure difference is as indicated below [17]:

(2) $$∆P_{c}=2\sigma \left(\frac{1}{R_{e}}-\frac{1}{R_{c}}\right)=2\sigma \left(\frac{cos\theta_{e}}{r_{e}}-\frac{cos\theta_{c}}{r_{c}}\right)$$

In the two equations, $∆P_{c}$ represents the capillary pressure difference, $∆P_{l}$ denotes the liquid pressure difference, $∆P_{v}$ stands for the vapor pressure difference, $R_{e}$ signifies the capillary radius in the evaporator section, $R_{c}$ represents the capillary radius in the condenser section, $\theta_{e}$ corresponds to the contact angle between the liquid and the inner wall in the evaporator section, and $\theta_{c}$ pertains to the contact angle between the liquid and the inner wall in the condenser section.

### 2.2. Heat Pipe Design Requirements

**Figure 2:**
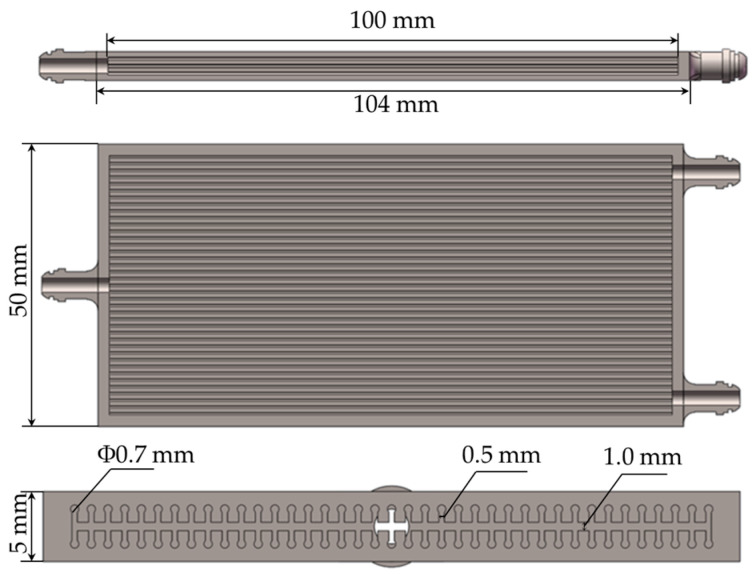
Three views of the heat pipe model.

The thermal pipe designed in this study was intended for use in the thermal control system of microsatellites. To allow for the efficient use of the limited internal space within the satellite and to control the mass of the thermal control system, the volume of the designed thermal pipe could not be too large. Therefore, it was necessary to choose a material for the pipe shell with a lower density. Additionally, a 3D printing device with a precision of 0.1 mm was employed for manufacturing to ensure the reliability of the thermal pipe. Considering these factors, we chose to use a common 3D printing material, aluminum alloy, which offers strong thermal conductivity and lower density. To align with the integrated design goals of SLM, a groove-type wick structure was adopted to provide capillary force. Common shapes for groove wicks include rectangular, rhomboidal, V-shaped, and Ω-shaped. In this study, we selected the Ω-shaped wick because of its characteristic of reducing the capillary force required for working fluid circulation and enhancing heat transfer performance [15]. The final dimensions of the thermal pipe are presented in Table 1 below: 

Following the mentioned requirements, the thermal pipe model shown in Figure 2 was created using the SolidWorks 2021R2 software.

## 3. Simulation of Flow and Heat Transfer in the Heat Pipe

### 3.1. Mathematical Model for Heat Pipe Simulation

This study employed a multiphase flow model to analyze the two-phase flow process of gaseous and liquid ethanol inside the heat pipe. The VOF (volume of fluid) multiphase flow model used is a common mathematical model for simulating internal flow within a heat pipe, known for its well-established mathematical foundation and practical experience. The expression to solve the phase volume fraction in the VOF (volume of fluid) model is as follows [18] and the units of variables in the formula are shown in Appendix A:

(3) $$\frac{1}{\rho_{q}}\left[\frac{\partial }{\partial t}\left(\alpha_{q}\rho_{q}\right)+\nabla \cdot \left(\alpha_{q}\rho_{q}{\vec{v}}_{q}\right)=S_{\alpha_{q}}+\sum_{p=1}^{n}\left({\dot{m}}_{pq}-{\dot{m}}_{qp}\right)\right]$$

In the equation, α represents the volume fraction of the corresponding phase, ρ is density, ${\dot{m}}_{qp}$ is the mass transfer term from phases *q* to *p*, ${\dot{m}}_{pq}$ is the mass transfer term from phases *p* to *q*, and $S_{\alpha_{q}}$ is the mass source term. When no flow is occurring, the entire right-hand side is equal to 0.

In addition to the multiphase flow model, the fluid flow inside the pipe also needed to account for the interaction between the fluid and the pipe wall. Therefore, it was necessary to introduce surface tension and wall adhesion models. Through the divergence theorem, surface tension is expressed as a volume force, and when there are only two phases in a control unit, surface tension can be represented as follows:

(4) $$F_{vol}=\sigma_{ij}\frac{\rho \kappa_{i}\nabla \alpha_{i}}{\frac{1}{2}\left(\rho_{i}+\rho_{j}\right)}$$

Here, κ represents the surface curvature of the heat pipe wall, and $\sigma_{ij}$ is the surface tension.

The wall adhesion model [19] was selected for the wall surface adhesion model. By adjusting the vertical vector near the wall, the surface curvature of the wall is adjusted. The actual curvature of the liquid surface is corrected by the unit surface normal vector, and the relationship between the normal vector and the contact angle is expressed as follows:

(5) $$\hat{n}={\hat{n}}_{w}cos\theta_{w}+{\hat{t}}_{w}sin\theta_{w}$$

In thermal pipe simulation, phase-change heat transfer and mass transfer are also involved. To calculate the mass transfer term $\dot{m}$ in the VOF model, this study used the Lee model, which is based on molecular dynamics principles. It equates the interface heat transfer to the mass transfer multiplied by the latent heat of a phase change, simulating phase-change heat and mass transfer sources. The Lee model equations are as follows [20,21,22]; Equations (6) and (7) calculate the evaporation mass transfer and condensation mass transfer, respectively:

(6) $$\dot{{\dot{m}}_{l\to v}=r_{l\to v}\rho_{l}\alpha_{l}\frac{T_{l}-T_{sat}}{T_{l}}}$$

(7) $$\dot{{\dot{m}}_{v\to l}=r_{v\to l}\rho_{v}\alpha_{v}\frac{T_{v}-T_{sat}}{T_{sat}}}$$

where *r* represents the mass transfer coefficient of evaporation and condensation, and *T* represents the temperature.

### 3.2. Simulation Results and Cloud Images

**Figure 3:**
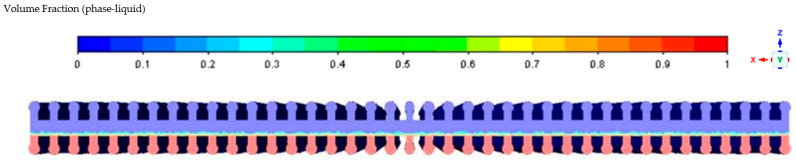
Gas–liquid two-phase distribution diagram of the initial state of the heat pipe.

In the simulation, to improve computational efficiency, the surfaces of the evaporation section and condensation section are set as constant flux boundary conditions. It is assumed that the heat sources and sinks are uniformly distributed on the surfaces of these two sections. Additionally, the middle section of the heat pipe is treated as an adiabatic section, where no heat exchange occurs. And since the inner volume of the three sealing plugs of the heat pipe only accounts for 1.87% of the total volume, which has a small impact on the heat transfer performance but will slow down the convergence of the simulation, the three sealing plugs are omitted from the simulation. In the initial state, the heat pipe is placed horizontally, with the lower half filled with liquid, as shown in Figure 3 below.

**Figure 4:**
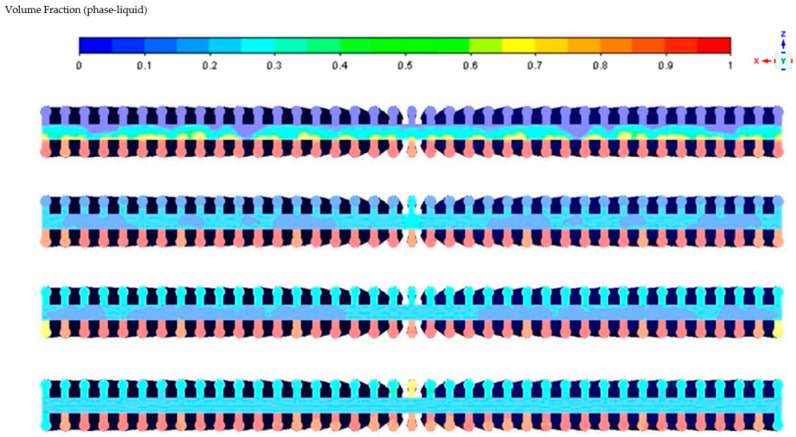
Changes in gas-liquid two-phase distribution at the cross section of the evaporation section.

As the temperature of the heat pipe increases, the liquid-phase working fluid in the evaporator section evaporates. It gradually rises from the bottom of the channels to the vapor chamber. Then, driven by the vapor pressure difference, it flows to the condenser section. In the condenser section, the condensed liquid is drawn back to the evaporator section due to capillary action. Eventually, this process results in both the upper and lower channel sections of the heat pipe having higher liquid-phase volume fractions than the liquid-phase volume fraction inside the vapor chamber. The evolution of this process is illustrated in Figure 4 below.

**Figure 5:**
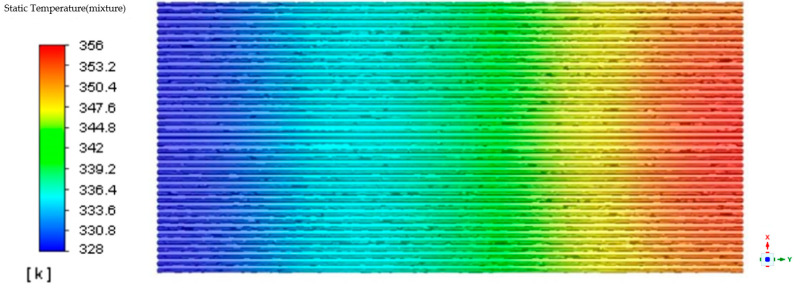
Heat pipe temperature distribution cloud diagram.

While calculating the two-phase volume fractions, the temperature distribution was also simulated. During the calculations, the time step was set to 0.01 s, and 20 iterations were performed for each time step. The total calculation time was ten minutes. In the stable state, the temperature in the evaporator section of the heat pipe was approximately 350 K, while the temperature in the condenser section was around 325 K. The resulting temperature distribution in the heat pipe at the stable state is illustrated in Figure 5 below.

Based on the simulation process, which captured the changes in the gas–liquid two-phase flow and the final stable temperature distribution, the simulation results closely aligned with the theoretical values. This indicates that the simulation effectively captured the flow and heat transfer phenomena inside the pipe.

## 4. The Influence of the Structural Parameters of the Heat Pipe on Heat Transfer Characteristics and Parameter Optimization

In this section, based on the simulation results obtained using ANSYS 2021R2 software, a multi-objective parameter optimization method was applied to analyze various structural parameters of the heat pipe and ultimately obtain the optimal heat pipe structure in terms of thermal conductivity.

The simulation results do not directly reflect the heat transfer capacity of the heat pipe. To visually represent the heat transfer characteristics of the heat pipe and facilitate subsequent optimization, the following equations (Equations (8) and (9)) were introduced to calculate the thermal resistance R [23] and the equivalent thermal conductivity $K_{eff}$ [24,25] of the heat pipe.

(8) $$R=\frac{∆T}{Q}$$

(9) $$K_{eff}=\frac{QL_{eff}}{A_{eff}∆T}$$

In the equations, where Q represents the heating power in the evaporator section, $L_{eff}$ is the effective length of the heat pipe, $A_{eff}$ is the effective cross-sectional area of the heat pipe, and ∆T denotes the temperature difference between the evaporator and condenser sections.

Modifications to the small satellite’s structure would slow down the development process. To avoid altering the internal layout of the satellite, the overall dimensions of the heat pipe remained unchanged during the optimization process. Additionally, the original metal heat plate used in the laboratory had a length of 110 mm. In the simulation and optimization process, the length of the heat pipe was kept constant at 104 mm, accounting for the space reserved for sealing plugs at both ends. The parameters selected for multi-objective regression optimization included the internal channel diameter (*d*), the height of the vapor cavity (*h*), and the width of the narrow gap (*w*). The heat pipe was manufactured using an EOS-M280 metal 3D printer with a precision of 0.1 mm. It was essential to consider that, if the printed dimensions were too small, they could lead to issues such as internal channel deformation and excessive porosity. After precision and print quality were taken into account, the optimization range for the channel diameter was set between 0.6 mm and 1.0 mm, the vapor cavity height ranged from 0.6 mm to 2.2 mm, and the narrow gap width was optimized within a range of 0.2 mm to 0.6 mm. Table 2 below outlines the parameter variations and their corresponding thermal resistance and equivalent thermal conductivity values.

By analyzing the results in the above table, a multiple-regression equation [26] for thermal resistance and equivalent thermal conductivity could be obtained:

(10) R=0.845+0.561d+0.299h−0.124w

(11) $$K_{eff}=278.712-53.156d-38.791h+34.313w$$

In multiple linear regression analysis, the *p*-values of parameters indicate the degree of influence of these parameters on the optimization objectives. The *p*-values for the three parameters are shown in Table 3 below. From the information in the table, it can be observed that the steam chamber height has a greater impact on the heat pipe’s thermal performance compared to the other two parameters.

For the feasibility of the final print and the stability of the heat pipe, the optimal values for each parameter were chosen as values greater than the minimum, Keep the final value to two decimal places. When the optimal solution was obtained, the values were as follows: the channel diameter d was 0.6 mm, the steam chamber height h was 0.6 mm, and the narrow slit width w was 0.48 mm. The optimal values for thermal resistance and equivalent thermal conductivity were 1.301 K/W and 240.014 W/(m·K), respectively.

Based on the optimized parameter values, simulations were conducted using the ANSYS model. The results showed that, at an input power of 15 W, the thermal resistance was 1.298 K/W, and the equivalent thermal conductivity was 246.533 W/(m·K). These values closely matched the calculated results. Compared to the heat pipe before optimization, the thermal resistance decreased by 10.5%, and the equivalent thermal conductivity increased by 11.6%. Compared to the traditional metal heat pipes used in microsatellites, the thermal resistance decreased by 38.5%, and the equivalent thermal conductivity increased by 62.19%.

## 5. Experimental Investigation of the Heat Transfer Characteristics of the Heat Pipe Made Using Selective Laser Melting Technology

### 5.1. A 3D Printing and Sealing Experiment with the Heat Pipe

**Figure 6:**
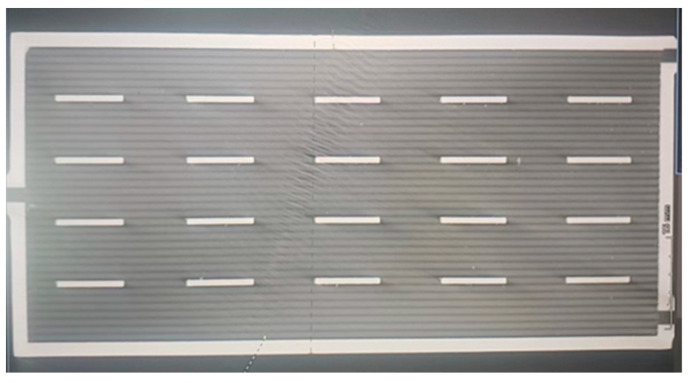
CT scan of the internal support structure of the heat pipe.

During the simulation, an idealized model of the heat pipe was used. However, during the printing process, due to the strength of the material and the impact of thermal stresses during processing, the channels inside the heat pipe were prone to collapsing and deforming. Therefore, modifications were made to the model via the addition of support rods inside some of the vapor chambers to stabilize the heat pipe’s structure. Figure 6 shows the internal support of the heat pipe after printing and forming.

The parameters of the 5024 aluminum alloy material used for metal powder printing are shown in Table 4 [27].

**Figure 7:**
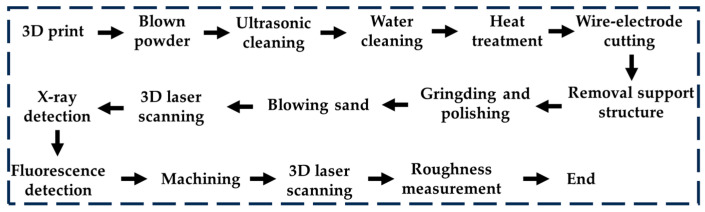
Post-processing process of SLM technology to produce heat pipes.

This experiment used SLM technology to produce heat pipes, using an M280 printer produced by German EOS company equipped with a 400 W light laser. The 3D model created using SolidWorks was first converted into an STL format file. After that, the model was sliced, discretized, and converted into a multilayer 2D model with a layer height of 0.03 mm, and the S-shaped scanning print path was used, which could improve the uneven stress distribution phenomenon caused by unidirectional scanning to a certain extent, and the post-processing process is shown in Figure 7. There will be powder residue inside a heat pipe printed using SLM technology, which needs to be removed before heat treatment; otherwise, it will condense inside the pipe and change the structure during heat treatment. After four hours of heat treatment, the internal stress of the heat pipe was basically eliminated, and the printing of the heat pipe was basically completed. Later, laser scanning was needed to test the printing quality of the heat pipe.

**Figure 8:**
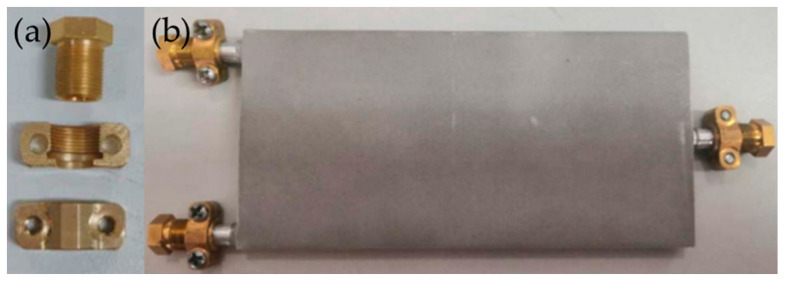
Sealing plugs of heat pipes and printed products of heat pipes: (**a**) sealing plugs and (**b**) heat pipe.

After printing, it was necessary to conduct a sealing test on the heat pipe. In this study, the device shown in Figure 8 was used to seal both ends of the heat pipe, and a bubble method was employed to check for leaks. This involved immersing the sealed heat pipe directly in warm water and observing it for some time. If the heat pipe was not sealed properly, the gas inside the pipe would have expanded and escaped, forming bubbles at the inlet and outlet of the heat pipe due to the increase in gas temperature. During the experiment, no bubbles were observed in the heat pipe, indicating that the heat pipe printed using SLM technology had good airtightness.

### 5.2. Quality Inspection of Heat Pipe Printing

**Figure 9:**
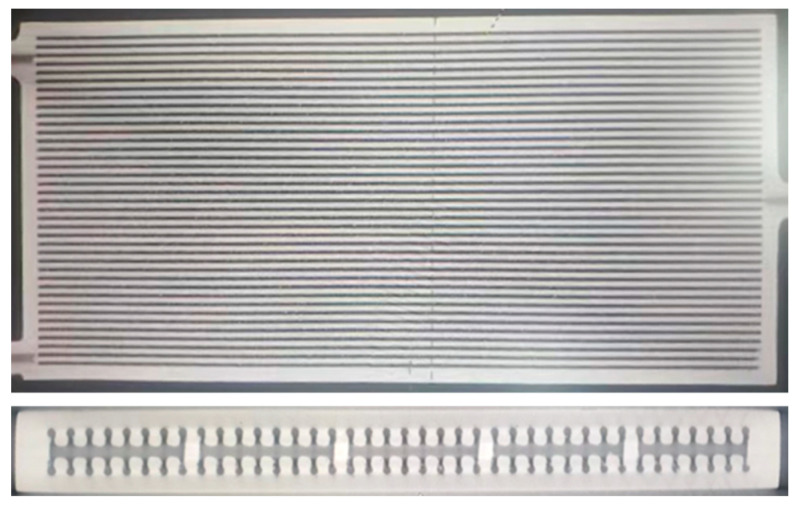
Internal scanning diagram of the heat pipe.

The industrial performance of the EOSM280 printer was good, and the printed heat pipe and the designed model were almost identical on the surface. However, due to the small size of the heat pipe and its application in precision instruments like microsatellites, high-performance requirements were placed on it [28]. However, selective laser melting (SLM) technology has some drawbacks, such as the challenging removal of powder residue and potential structural deformations [29,30]. To ensure that the printed products are fit for use, before proceeding with further experiments, we used a CT scanner to inspect its internal structure [31,32]. The inspection results are depicted in the following figure, Figure 9. According to the structure of the picture, it could be found that the channel structure of the heat pipe was the same as the designed structure, and the middle part had not undergone deformation, indicating that SLM technology achieves good performance in producing small heat pipes and could be used for the next step of our experiments.

### 5.3. Heat Pipe Vacuum Environment Experiment

**Figure 10:**
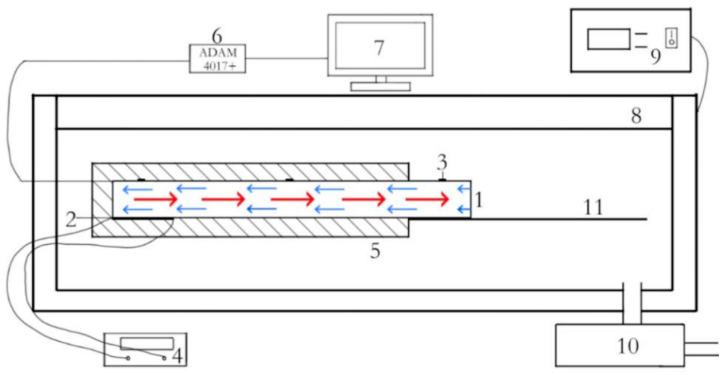
Vacuum environment for the heat pipe experimental device: (1) heat pipe; (2) electric heater; (3) thermocouple; (4) power supply; (5) thermal insulation, polyethylene clinker foam; (6) data acquisition module; (7) computer; (8) vacuum tank; (9) vacuum gauge; (10) air extraction pump; and (11) heat sink.

**Figure 11:**
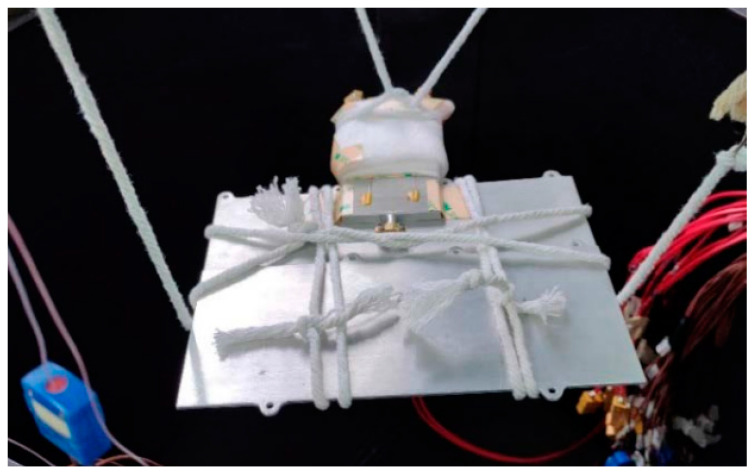
Physical diagram of the vacuum device.

As the heat pipe was primarily intended for use in the space environment within microsatellites, the experiments needed to be conducted in a vacuum. According to the experimental setup, the heat pipe was suspended inside a vacuum chamber, as illustrated in Figure 10, for testing. Figure 11 provides a physical representation of the experimental setup.

The experimental process involved first filling the heat pipe and ensuring its seal. Then, temperature sensors (thermocouples) were connected to both ends of the heat pipe for temperature data collection. The heat pipe was suspended inside a vacuum chamber. The vacuum chamber was sealed, and its internal air was removed using a vacuum pump. The power source was turned on to heat the heating section of the heat pipe. The temperature data collected using the sensors were observed. The experiment concluded once the temperatures had stabilized.

**Figure 12:**
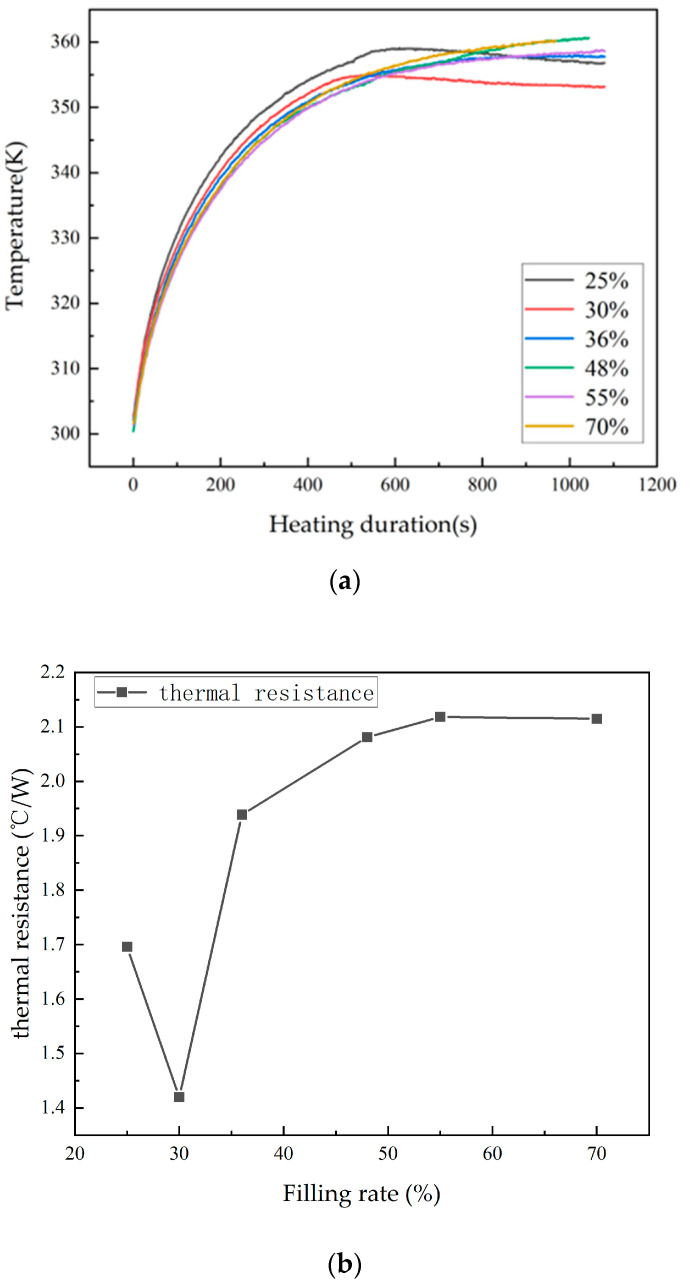
Temperature variation curve and thermal resistance variation diagram of heat pipes under different liquid filling rates: (**a**) temperature changes under different filling rates and (**b**) the thermal resistance of heat pipes under stable conditions with different filling rates.

First, experiments were conducted to investigate the effect of the fill ratio on the heat transfer performance of the heat pipe. The proportion of the working fluid inside the heat pipe varied, and the heat pipe was heated for the same duration with a 15 W heating power. Temperature data from the heat pipe were collected, resulting in the curve shown in Figure 12. When comparing the data, it is evident that, among heat pipes with different fill ratios, the one with a 30% fill ratio had the lowest temperature, indicating the best heat dissipation capability and the highest heat transfer performance.

**Figure 13:**
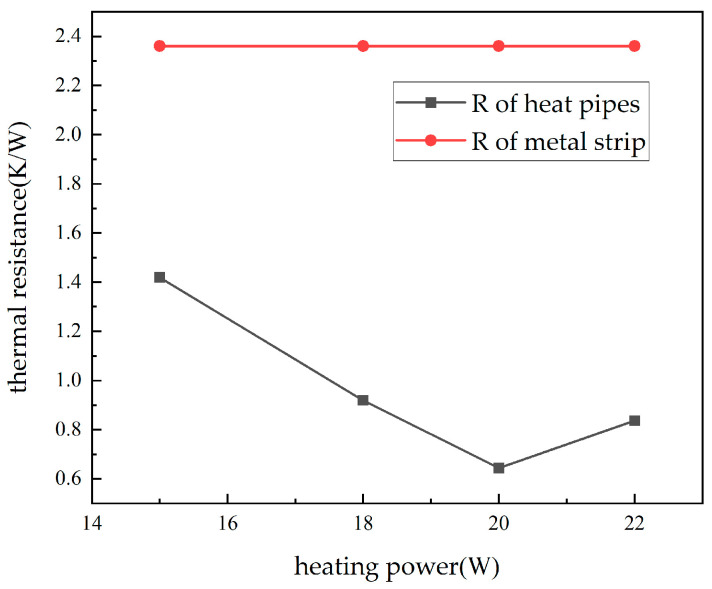
Thermal resistance of heat pipes and metal strips at different powers.

The next experiment compared the heat transfer performance of the heat pipe with that of a metal rod. The temperatures of the heat pipe and the metal rod were measured when they reached a stable operating state at different power levels. The thermal resistance values were then calculated, resulting in the thermal resistance variation chart shown in Figure 13. It can be observed that, at all heating power levels, the thermal resistance of the heat pipe was lower than that of the metal rod, indicating a significant improvement in heat transfer capability compared to traditional heat conduction rods in this study.

**Figure 14:**
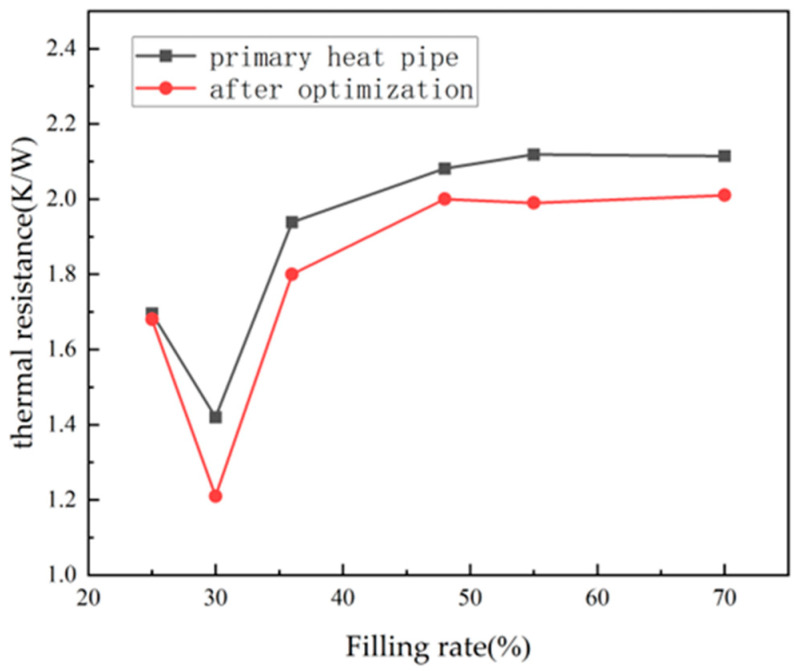
Comparison of thermal resistance of heat pipes before and after optimization under different liquid filling rates.

Finally, the optimized heat pipe was 3D-printed and subjected to thermal performance testing experiments. The experimental approach involved measuring and calculating the thermal resistance values of the new heat pipe at different filling ratios and comparing the results with the original heat pipe. The resulting curve in Figure 14 shows that the thermally optimized heat pipe significantly outperformed the pre-optimized one in terms of thermal conductivity. Across various filling ratios, the thermal resistance values of the optimized heat pipe had notably decreased. For the optimal working condition with a 30% filling ratio, the thermal resistance had decreased by 15%, indicating a significant improvement in heat transfer capacity and demonstrating the feasibility of the optimization method used.

## 6. Conclusions

In this study, a small heat pipe manufactured using SLM technology was designed to meet the temperature control requirements of microsatellite applications. The heat pipe utilized the principle of the convective heat transfer of the work material, while the original heat conduction strip simply relied on heat conduction and radiation heat dissipation; the vaporization–liquefaction process of the work material and capillary action can significantly improve the heat transfer efficiency, significantly improving the heat dissipation capacity in the satellite. In order to verify the feasibility of the design, the simulation calculations, print fabrication, and experimental validation of the heat pipe were carried out.

SolidWorks and ANSYS were used for modeling and simulation to assess the heat pipe’s fluid flow and heat transfer capabilities. The simulation results indicated the excellent heat transfer performance of the heat pipe. Building upon these results, the geometric structure of the heat pipe was optimized.

Multiple parameters, including the heat pipe ratio, channel diameter, heat pipe vapor chamber height, and narrow gap width, were subjected to multi-objective regression optimization. The geometric structure that yielded the optimal heat transfer performance was determined. The optimized heat pipe demonstrated a 10.5% reduction in thermal resistance and an 11.6% increase in equivalent thermal conductivity compared to the original design. When compared to the traditional heat-conducting bars used in microsatellites, the optimized heat pipe exhibited a 38.5% reduction in thermal resistance and a 62.19% increase in equivalent thermal conductivity.

To validate the reliability of the simulation results, SLM was employed to fabricate the heat pipes before and after optimization. Experimental tests were conducted to assess the thermal conductivity of these different heat pipes. The experiments revealed that the thermal resistance of the optimized heat pipe decreased by 15%, leading to a significant improvement in heat transfer performance. This confirmed the reliability of the simulation results and demonstrated the feasibility of 3D-printed heat pipes.

When printing the heat pipes, an erroneous estimation of the material strength of the aluminum alloy led to issues. Although the outer shells of the early printed samples were compliant, they invariably experienced internal channel collapses and bottom warping. This situation resulted in resource wastage, impeded the progress of the experiments, and, in turn, delayed the project. The incorporation of support structures subsequently addressed this problem and provided invaluable insights for future SLM experiments.

The discrepancies between the experimental and computational results can be attributed to the absence of support structures in the simulation. During the optimization process, support structures were not included within the heat pipe model in order to improve efficiency. In contrast, the experimental heat pipe did incorporate support rods, leading to variations in thermal resistance and differences compared to the simulation results when structural changes were introduced. And due to the limitations of printer accuracy, the printed product cannot be as accurate as the simulation results to 0.01 mm, with a slight size deviation. This deviation increases the error between the simulation and the experimental results. So, after this study, how to enhance printing accuracy and improve the quality of printing results is also a planned research direction.

The high designability of SLM technology has provided many possibilities and options for the internal structure of small satellites [33]. This study has proven that the performance of heat pipes produced using SLM technology can meet practical requirements. In the future, plans are made to integrate the design of heat pipes with storage tanks and pipelines inside a satellite, achieving an integrated structure of storage, heat dissipation, and mass transfer, which can further shorten the production cycle and cost of the satellite.

## Figures and Tables

**Table 1:** Structural parameters of the heat pipe model.

Parameter	Size/mm
Total length	104
Heat pipe width	50
Heat pipe height	5
Channel diameter	0.7
Narrow slit width	0.5
Steam chamber height	1

**Table 2:** Uniform design table and simulation analysis results.

Serial Number	d/mm	h/mm	w/mm	R/K/W	$K_{eff}$/W/(m·K)
1	0.6	1.0	0.5	1.425	224.614
2	0.7	1.8	0.4	1.721	185.974
3	0.8	0.6	0.3	1.430	223.776
4	0.9	1.4	0.2	1.750	182.857
5	1.0	2.2	0.6	1.990	160.804

**Table 3:** *p*-values of various parameters in multiple regression analysis.

Parameter	*p*-Value of Thermal Resistance	*p*-Value of Equivalent Thermal Conductivity
Channel diameter d	0.0525	0.0527
Steam chamber height h	0.0285	0.0209
Gap width w	0.2282	0.0814

**Table 4:** Aluminum alloy material parameters.

Material	Density/kg/m^3^	Yield Strength Rp0.2/MPa	Tensile Strength Rm/MPa
Aluminum alloy 5024	2660	482	527

## Data Availability

The data and results involved in this study have been presented in detail in this paper.

## Appendix A

Here are the units of each variable mentioned in the text:

$$∆P_{c},\;∆P_{l},\;∆P_{v}——\mathrm{MPa}$$

$$R_{e},\;R_{c}——\mathrm{mm}$$

$$\rho ——\mathrm{kg}/m^{3}$$

$${\dot{m}}_{qp},\;{\dot{m}}_{pq},\;{\dot{m}}_{l\to v},\;{\dot{m}}_{v\to l}——\mathrm{kg}/s$$

κ——1/mm

$$T_{l},\;T_{v},\;T_{sat}——K$$

$$L_{eff}——\mathrm{mm}$$

$$A_{eff}——{\mathrm{mm}}^{2}$$

$$K_{eff}——W/m\cdot K$$

## References

[1] Varatharajoo R., Kahle R., Fasoulas S. (2010). Approach for Combining spacecraft attitude and thermal control systems. J. Spacecr. Rocket..

[2] Swanson T.D. Thermal Control Technologies for Complex Spacecraft. Proceedings of the 13th International Heat Pipe Conference.

[3] Khrustalev D., Faghri A. (1994). Thermal analysis of micro heat pipe. J. Heat Transf..

[4] Khrustalev D., Faghri A. (1995). Thermal characteristics of conventional and flat miniature axially grooved heat pipe. J. Heat Transf..

[5] Chen Y., Zhang C., Shi M., Wu J., Peterson G.P. (2009). Study on flow and heat transfer characteristics of heat pipe with axial “Ω”-shaped microgrooves. Int. J. Heat Mass Transf..

[6] Zhang C., Chen Y., Shi M., Peterson G.P. (2009). Optimization of heat pipe with axial “Ω”-shaped micro grooves based on a niched Pareto genetic algorithm (NPGA). Appl. Therm. Eng..

[7] Yao F., Bian N., Xia Y., Chen W., Zhang R. (2021). Thermal performance of an axially grooved heat pipe subjected to multiple heating sources. Microgravity Sci. Technol..

[8] Annamalai N.R.F. (2012). Experimental investigation and computational fluid dynamics analysis of a wick heat pipe. Int. J. Therm. Sci..

[9] Szczukiewicz S., Magnini M., Thome J.R. (2014). Proposed models, ongoing experiments, and latest numerical simulations of microchannel two-phase flow boiling. Int. J. Multiph. Flow.

[10] Rabiee R., Rajabloo B., Désilets M., Proulx P. (2019). Heat transfer analysis of boiling and condensation inside a horizontal heat pipe. Int. J. Heat Mass Transf..

[11] Yasuda Y., Nabeshima F., Horiuchi K., Nagai H. (2023). Comparison of heat-transfer performance of a flat-plate pulsating heat pipe based on heating orientation and cross-sectional shape of the pipe. Mech. Eng. J..

[12] Enke C., Júnior J.B., Vlassov V. (2021). Transient response of an axially-grooved aluminum-ammonia heat pipe with the presence of non-condensable gas. Appl. Therm. Eng..

[13] Anand A.R. (2019). Analytical and experimental investigations on heat transport capability of axially grooved aluminium-methane heat pipe. Int. J. Therm. Sci..

[14] Shen C., Zhang Y., Wang Z., Zhang D., Liu Z. (2021). Experimental investigation on the heat transfer performance of a flat parallel flow heat pipe. Int. J. Heat Mass Transf..

[15] Wang G., Quan Z., Zhao Y., Wang H. (2020). Effect of geometries on the heat transfer characteristics of flat-plate micro heat pipes. Appl. Therm. Eng..

[16] Chen H., Mo H., Wan Z., Huang S., Wang X., Zhu H. (2020). Thermal performance of a boiling and condensation enhanced heat transfer tube—Stepped lattice finned tube. Appl. Therm. Eng..

[17] Brackbill J.U., Kothe D.B., Zemach C. (1992). A continuum method for modeling surface tension. J. Comput. Phys..

[18] Alizadehdakhel A., Rahimi M., Alsairafi A.A. (2010). CFD modeling of flow and heat transfer in a thermosyphon. Int. Commun. Heat Mass Transf..

[19] Vachaparambil K.J., Einarsrud K.E. (2019). Comparison of surface tension models for the volume of fluid method. Processes.

[20] Pu L., Li Q., Shao X., Ding L., Li Y. (2019). Effects of tube shape on flow and heat transfer characteristics in falling film evaporation. Appl. Therm. Eng..

[21] Tan Z., Cao Z., Chu W., Wang Q. (2023). Improvement on evaporation-condensation prediction of Lee model via a temperature deviation based dynamic correction on evaporation coefficient. Case Stud. Therm. Eng..

[22] Jeong C.H., Lee H.J., Choi C.K., Lee S.H. (2021). Selective evaporation rate modeling of volatile binary mixture droplets. Int. J. Heat Mass Transf..

[23] Chen J., Xu X., Zhou J., Li B. (2022). Interfacial thermal resistance: Past, present, and future. Rev. Mod. Phys..

[24] Liu N., Li N., Li G., Song Z., Wang S. (2022). Method for evaluating the equivalent thermal conductivity of a freezing rock mass containing systematic fractures. Rock Mech. Rock Eng..

[25] Cao D., Xing C., Qin Y., Lou G., Shen X. (2023). Mathematical model on equivalent thermal conductivity coefficient of foamed concrete. Proceedings of the Second International Conference on Statistics, Applied Mathematics, and Computing Science (CSAMCS 2022).

[26] Zeng L., Wu W., Li Z. (2023). Research on multiple regression linear prediction model of 3D printing process parameters. Proceedings of the Third International Conference on Mechanical, Electronics, and Electrical and Automation Control (METMS 2023).

[27] Mondolfo L.F. (2013). Aluminum Alloys: Structure and Properties.

[28] Mushtaq R.T., Iqbal A., Wang Y., Khan A.M., Petra M.I. (2023). Advancing PLA 3D Printing with Laser Polishing: Improving Mechanical Strength, Sustainability, and Surface Quality. Crystals.

[29] Trenfield S.J., Xu X., Goyanes A., Rowland M., Wilsdon D., Gaisford S., Basit A.W. (2023). Releasing fast and slow: Non-destructive prediction of density and drug release from SLS 3D printed tablets using NIR spectroscopy. Int. J. Pharm. X.

[30] Vashist V., Oldal I., Szakal Z. (2023). Analysis of mechanical properties of 3D printed metallic specimens of aluminum (CL31) bronze (CL80) material manufactured at varying laser scanning speed. J. Phys. Conf. Series. IOP Publ..

[31] Sommacal S., Matschinski A., Holmes J., Drechsler K., Compston P. (2023). Detailed void characterisation by X-ray computed tomography of material extrusion 3D printed carbon fibre/PEEK. Compos. Struct..

[32] Ning R., Wang D., Zhao J., Rong L., Wang Y. (2023). High accuracy terahertz computed tomography using a 3D printed super-oscillatory lens. Opt. Lasers Eng..

[33] Lee S.H., Kim H.W., Park H.J. (2023). Integrated design of micro-fibrous food with multi-materials fabricated by uniaxial 3D printing. Food Res. Int..

